# Exploratory Research on Sweetness Perception: Decision Trees to Study Electroencephalographic Data and Its Relationship with the Explicit Response to Sweet Odor, Taste, and Flavor

**DOI:** 10.3390/s22186787

**Published:** 2022-09-08

**Authors:** Elena Romeo-Arroyo, Javier Soria, María Mora, Francisco Laport, Aitor Moreno-Fernandez-de-Leceta, Laura Vázquez-Araújo

**Affiliations:** 1 BCC Innovation, Technology Center in Gastronomy, Basque Culinary Center, 20009 Donostia-San Sebastián, Spain; 2 Basque Culinary Center, Faculty of Gastronomic Sciences, Mondragon Unibertsitatea, 20009 Donostia-San Sebastián, Spain; 3 i3B, Ibermática Institute of Innovation, Gipuzkoa Technology Park, Paseo Mikeletegi, 5, 20009 Donostia-San Sebastián, Spain; 4 CITIC Research Center, University of A Coruña, 15008 A Coruña, Spain

**Keywords:** EEG, sucrose, clustering algorithms, sensory modality discrimination

## Abstract

Using implicit responses to determine consumers’ response to different stimuli is becoming a popular approach, but research is still needed to understand the outputs of the different technologies used to collect data. During the present research, electroencephalography (EEG) responses and self-reported liking and emotions were collected on different stimuli (odor, taste, flavor samples) to better understand sweetness perception. Artificial intelligence analytics were used to classify the implicit responses, identifying decision trees to discriminate the stimuli by activated sensory system (odor/taste/flavor) and by nature of the stimuli (‘sweet’ vs. ‘non-sweet’ odors; ‘sweet-taste’, ‘sweet-flavor’, and ‘non-sweet flavor’; and ‘sweet stimuli’ vs. ‘non-sweet stimuli’). Significant differences were found among self-reported-liking of the stimuli and the emotions elicited by the stimuli, but no clear relationship was identified between explicit and implicit data. The present research sums interesting data for the EEG-linked research as well as for EEG data analysis, although much is still unknown about how to properly exploit implicit measurement technologies and their data.

## 1. Introduction

Several studies have suggested that food liking and choice depend on different factors, including the product properties, consumer’s characteristics, and the context of consumption, a set of variables that have a significant impact on food perception [1,2]. Several authors have reported innate food-associated preferences, such as a positive predilection for sweet tastes and flavors, but these instinctive responses may be modified through the experience of growing up in a specific dietary and culinary culture: the human brain learns to relate sensory stimuli that it has previously found together in different foods [3,4,5]. Spence [6] reported that olfactory stimuli that have been regularly paired with sweet-, bitter-, salty-, or sour-tasting foods could enhance the associated taste quality, even when presented at a sub-threshold level. Considering this idea of cross-modal associations, recent research has demonstrated that the addition of a sweet aroma (e.g., vanilla) can be used to increase the perception of sweetness of different foods, and therefore to significantly reduce the amount of sugar added in their formulas [7,8]. Although promising results are increasing interest in this field, much is still unknown about the mechanisms behind cross-modal responses and the sweetness perception process, and research is needed to promote healthy food choices in a society with a growing demand for sweet products. A better understanding of brain responses to different modalities of sweet stimuli (sweet smell, sweet taste) could be useful in designing new foods and experiences that trigger specific positive reactions in consumers, and therefore in reducing sugar consumption among the population.

It has been suggested that the use of electroencephalography (EEG) techniques can be useful for marketing and sensory sciences because the activation of certain regions of the brain can offer information about people’s unfiltered response, providing evidence associated with the emotions and unconscious feelings of consumers [9]. The stimulation of gustatory, olfactory, and trigeminal receptors while eating triggers a cascade of reactions that will ultimately cause a neuronal activation in specific regions of the brain [10]. Part of this neuronal activation can be measured using EEG techniques, which are based on the reading of the electrical potentials detected in the scalp of the person, and that will be registered in electroencephalograms. Using EEG allows to collect immediate processing data of the presented stimuli (e.g., a taste or an odorant) to be gathered from fluctuations in brain signal frequencies [11].

Various methodologies have been reported to study the EEG as a tool to record responses to taste and olfactory stimuli. Andersen et al. [12] used a panel with 24 volunteers to study the grand-average evoked potentials associated with different sweeteners (sucrose, aspartame, and acesulfame K solutions), which were placed in the center of the tongue of the volunteers using a programable pump. These authors recorded 3 s responses and repeated the recording procedure 62 times (average) per volunteer. Mouillot et al. [13] assessed the gustatory evoked potentials (GEPs) elicited by different sweeteners in 20 subjects, also collecting the self-reported response to the stimuli and looking for relationships between conscious and unconscious responses. Results indicated that the brain response to different sweetness was significantly different by substance, although the self-reported response of volunteers was not useful to classify the sweet substances. Both studies focused on different gustatory stimuli, but no other sweet stimuli were assessed (e.g., sweet odors). Crouzet, Busch, and Ohla [14] linked patterns of neuronal response of 16 volunteers with self-reported taste perception using electroencephalographic data: the tastes that consumers discriminated with higher clarity were those that generated a stronger and more differential brain signal. These authors used a four-alternative forced-choice taste discrimination task followed by the analyses of the global field power and global map dissimilarity of the electrophysiological brain responses. Regarding olfactory stimuli, different electrophysiological studies have been conducted, showing the complexity of the matter and the need for including self-reported responses to better understand the whole cognitive process of olfaction [15]. Placidi et al. [16] studied brain response to remembering unpleasant odors, focusing on the gamma and alpha bands (32–42 Hz and 8–30 Hz, respectively) and the P4, C4, T8, and P8 channels, but no odorants or volatile compounds were assessed in this study. Kroupi et al. [17] investigated the alterations in brain activity during the stimulation with six hedonically-different odors and showed that odor pleasantness could be predicted with EEG data when a subject-specific classifier was trained, and that some generic patterns were observed when subject-independent analysis was performed. Although different investigations have been conducted to better understand brain activity while exposed to an odorant, none of them considered that some aromas can share descriptors and responses with taste stimuli (e.g., sweetness) or researched potential common mechanisms.

Although several studies have been conducted to investigate the non-conscious response to different sweet tastes [12,13] and to different odorants [15], to date, no studies have been published comparing the EEG response to sweet-taste and sweet-aromatics in food matrices. The present study was developed to explore the neurological response processes associated with the sucrose-sweet taste and a sweet-related aroma (vanillin) exposure in food matrices, and to study whether the implicit response (EEG) could be correlated with the explicit response. In addition, a sample including sucrose and vanilla was added to explore the response to sweet flavor. Different flavors were also added in the experimental design (dimethyl sulfide, cayenne) to determine if the classifications of sweet taste/aroma were different from those of other taste/aroma categories.

## 2. Materials and Methods

### 2.1. Participants

Eighteen healthy subjects (age mean = 29.6 ± 5.1; 10/8 women/men, respectively) participated in the present study. The number of volunteers was similar to the one reported by other authors studying EEG response [18]. Participants were recruited via the Basque Culinary Center (BCC, Donostia-San Sebastián, Spain) consumers’ database. Explicit and implicit recordings were performed on different days; therefore, participants attended BCC’s dependences twice. The study design was presented to, assessed, and approved by the Basque Country Drug Research Ethics Committee (reference number: PS2019050), and was conducted according to the Declaration of Helsinki. All subjects were properly informed about the research protocol and signed an informed consent to participate in the study. Only non-smoker volunteers with self-reported normal taste perception participated in the study. Participants were asked to not drink coffee/tea or eat at least 2 h before the study.

### 2.2. Samples

Although the aim of the study was to explore the EEG responses to sweet taste, sweet aroma, and sweet flavor, 2 non-sweet stimuli were included in the paradigm to test if the sweet category could be clearly differentiated from other odor/flavor categories and had common response patterns. Therefore, different stimuli, olfactory and gustatory, were chosen to determine the implicit and explicit responses.

For the olfactory stimuli, 2 volatile compounds representing 2 different aromatic categories were selected: (1) vanillin (97%, Merck, MO, USA), an organic compound present in vanilla, representing sweet aromatics; and (2) dimethyl sulfide (DMS) (99%, Merck, MO, USA), a sulfurous organic compound that is related to canned corn or cooked vegetables [19]. Odor stimuli were presented in 2 mL microcentrifuge tubes containing small cotton balls impregned with 600 μL of the corresponding diluted chemical compounds (1000 mg L^−1^). Stimuli were prepared ensuring a suprathreshold level; odor threshold for vanillin and DMS are 53 and 0.84 μg L^−1^, respectively [20].

For the taste and flavor stimuli, to favor a gradual release of the flavor without chewing or significantly moving the mouth, several food matrices were proposed and tested with the advice of EEG technology experts (ANT Neuro, Hengelo, The Netherlands). Chewing and/or swallowing movements could generate much noise in the EEG signal, therefore making EEG data analysis difficult. A marshmallow-like matrix was chosen (1.5 cm cubes) because it maintained a soft texture that melted slowly in the mouth without the need to chew. Three different formulas were prepared: a sweet-taste marshmallow with 44% sucrose, a sweet-flavor marshmallow with 27% of sucrose and 0.75% vanillin essence (Eurovanille, Gouy-Saint-André, France), and a non-sweet flavor marshmallow with a 1% of cayenne powder. Although cayenne contains capsaicin, an irritant that may trigger different brain reactions [21], this ingredient was chosen to ensure a completely different reaction to sweet stimuli.

**Figure 1:**
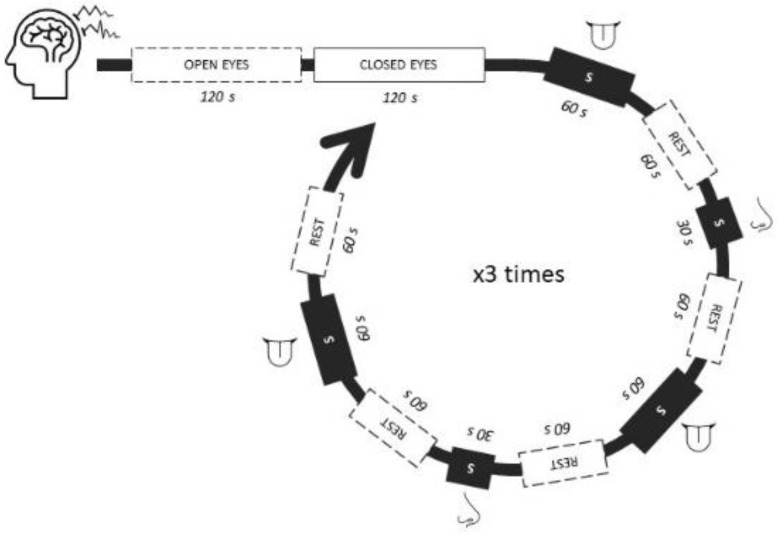
Example of one of the randomized paradigms during the EEG recordings. Legend: open and closed eyes periods were common in all participants; the rest of the sequence was presented in at least 3 different random orders to each participant.

Therefore, the final paradigm included a set of 2 odor and 3 flavor stimuli which were tested at least 3 times, in random order and blinded condition (participants were not informed of the descriptor associated with the stimuli they were about to assess, although they could guess its nature—smell/taste—when receiving the instruction to open their mouths to assess the taste/flavor ones), by each volunteer during the EEG and self-reported recording sessions (Figure 1).

### 2.3. Implicit Response

The experiment was held in an isolated dark room to prevent external cues interferences (visual and acoustic). During recording, volunteers were sitting in a comfortable armchair, and received instructions on how they should behave to test each type of sample: odor samples (opened microcentrifuge vials) were placed 2 cm from the volunteer’s nostrils for 30 s while deeply but normally breathing; for the marshmallow (msm) samples, volunteers had to keep the corresponding sample in the center of their tongue for 60 s, time enough for the taste and flavor release, and also to collect a segment of signal long enough to allow cutting some seconds linked to heavy movements at the beginning of the task (e.g., closing the mouth) if needed. Participants were instructed not to chew or swallow the msm samples, but to let them melt in their mouths. Before repeating the cycle of the set of samples (Figure 1), the volunteers were allowed to rest for at least 5 min and drink water, avoiding saturation or desensitization of their senses. Depending on their own reported state of saturation and tiredness, volunteers repeated the cycle from 3 to 5 times.

**Figure 2:**
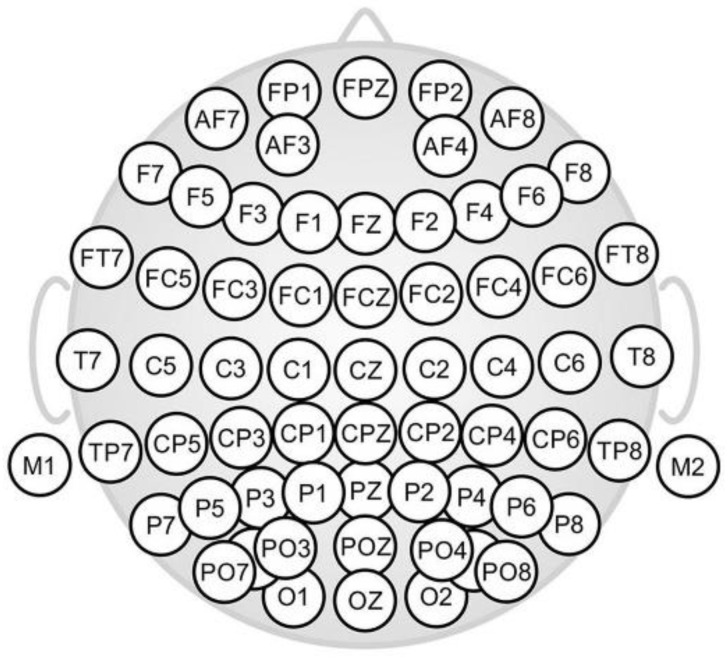
The 64-channel electrode cap topographic layouts with extended 10–20 layout (shows nose on top). Figure retrieved from Di Fronso et al. [22].

Implicit response was measured using a 64-channel dry electrode cap (ANT Neuro, Netherlands) with electrodes positioned following the 10–20 system (Figure 2); ground and reference channels were placed on the mastoid bones. Channel impedances and other technical recommendations indicated by the manufacturer (ANT Neuro, Netherlands) were followed prior to recording; sampling rate was 1024 Hz. Before starting with the samples, volunteers were left resting in the room for few minutes, getting used to the space and the cap, and then a 2 min open eyes segment and 2 min closed eyes segment were recorded. Then, the EEG signal was recorded before (baseline), during (epochs), and after the exposure to the different stimuli, always in closed-eyes mode. Resting periods of at least 1 min were left between samples, when participants were allowed to drink water and rinse their mouths. Each stimulus was presented to the subject at least 3 times in a random order.

#### EEG Data Analysis

To characterize the differences among signal patterns related to sweet odor, taste, and flavor stimuli, a workflow was developed and standardized from the recorded EEG data amplitude in the time domain. Datasets were converted from ANT-neuro to EEGLab format using Matlab EEGLab (MathWorks, Inc., Natick, MA, USA) and, consecutively, the ‘mne’ Python library was used for data processing [23]. To reduce data volume, sampling rate was reduced from 1024 Hz to 512 Hz. Then, noise reduction was achieved by filtering data with a high bandpass of 1 Hz and a low-pass filter with a cut-off frequency below the power line (50 Hz). Differently from other investigations, all potential cognitive activity involved in the perception processes was included in the data analyses, as suggested by Kosters [24] (beta, 14–30 Hz; alpha, 8–13 Hz; theta, 4–7 Hz; and delta, 1–3 Hz). 

Subsequently, an additional artifact removing step was performed by artifact subspace reconstruction (ASR), a statistical anomaly detection method that assumes that non-brain signals can be detected based on their deviant statistical properties [25]. The maximum acceptable standard deviation used for artifact removal was set to 20 for each 0.5 s window over all recorded signals. Then, to reduce any temporal drift unrelated to the experimental question, a baseline correction was performed using a mean subtraction procedure where the average voltage values of each electrode were calculated and then subtracted from the whole signal. Finally, a total of 390 labeled epochs were extracted for associated labels at each timestamp (vanilla ‘sweet-odor’, vanilla msm ‘sweet-flavor’, DMS ‘non-sweet odor’, cayenne msm ‘non-sweet flavor’, basic msm ‘sweet-taste’; approximately 30 s for odor stimuli and 60 s for taste and flavor stimuli; 18 individuals; at least 3 times each stimulus). Data were grouped by event and statistical descriptors were calculated by epochs: average, median, standard deviation, variance, maximum, minimum, skewness, and kurtosis.

Once the EEG signals were processed and the final dataset obtained, a data characterization was carried out using unsupervised clustering techniques. The idea behind the use of clustering was to find differential patterns in the signals able to distinguish either between studied stimuli or even between subjects. To eliminate any type of bias in the parameterization of the clustering algorithms, and therefore in the finding of patterns, a hyperparameter optimization was performed by grid search, which is an exhaustive searching through a manually specified subset of the hyperparameter space of a clustering algorithm. The evaluated hyperparameters were distance metric, number of clusters, and clustering algorithm, giving a total of 216 different scenarios. The selection of the best scenarios was carried out based on clustering performance metrics such as connectivity within clusters, the Davies–Bouldin index, the Dunn index, the silhouette width, and the balance between clusters by Monte Carlo cross-entropy ranking aggregation.

To determine and differentiate signal patterns related to olfactory and gustatory stimuli, descriptive modeling was carried out using rule-based decision trees which allowed to characterize the different smells and flavors. This analytical technique has been previously used to classify EEG signals [26]. A 100X cross-validation was used to test the descriptive models’ performance.

### 2.4. Explicit Response

One of the objectives of the study was to identify the potential relationship between the implicit and the explicit response to the sweet stimuli. Therefore, in addition to the EEG recording, a questionnaire was used to collect self-related responses in a second tasting session, conducted approximately one week after the EEG recording session. During this session, ratings on overall liking (9-point hedonic scale; 1 = extremely dislike, and 9 = extremely like) and the emotions elicited by the samples using the SEFrOS lexicon reported by Romeo-Arroyo, Mora, and Vázquez-Araújo [27] (15 cm linear scale with anchors from ‘not at all’ to ‘very intense’), were asked. Explicit response sessions were conducted in a taste room with individual booths and controlled environmental conditions (21 ± 2 °C; 55 ± 5% RH); the illumination was a combination of natural and nonnatural light (fluorescent).

#### Explicit Data Analysis

Results of the explicit responses to each emotional category elicited by the samples and liking were inputted in ANOVA tests as dependent variables and using ‘sample’ as a factor. Post hoc tests were conducted using Tukey’s HSD. In addition, a principal component analysis (PCA) was performed on the average ratings of each emotional category to visualize not only the statistical difference of the groups, but also their similarity in the vectorial space. Liking was used as a supplementary variable in the PCA analysis. In addition, to study the implicit–explicit response relationship, an ANOVA test was conducted using the factor ‘cluster’ from the hierarchical clustering analysis of the implicit response; it is important to mention that these results should be considered tentative because the number of individuals in each cluster was not equivalent. Statistical analyses of the explicit response were performed using XLSTAT [28].

## 3. Results

### 3.1. Implicit Response

The key performance indicator (KPI) of data after all filtering, artifacts removal, and processing was 98.8%, indicating a high quality of data. The KPI calculation included a multidimensional approach in which data completeness, consistency, accuracy, correctness, and outliers influence were ranked from 1 to 100 and averaged.

Data were modeled with three different clustering techniques, and the performance of each of them was assessed using eight different distance measurements and different distributions of the number of clusters; a total of 216 scenarios were tested. The best model was identified by applying the Monte Carlo method searching, which showed that the best clustering method was the hierarchical cluster with Canberra distance.

**Figure 3:**
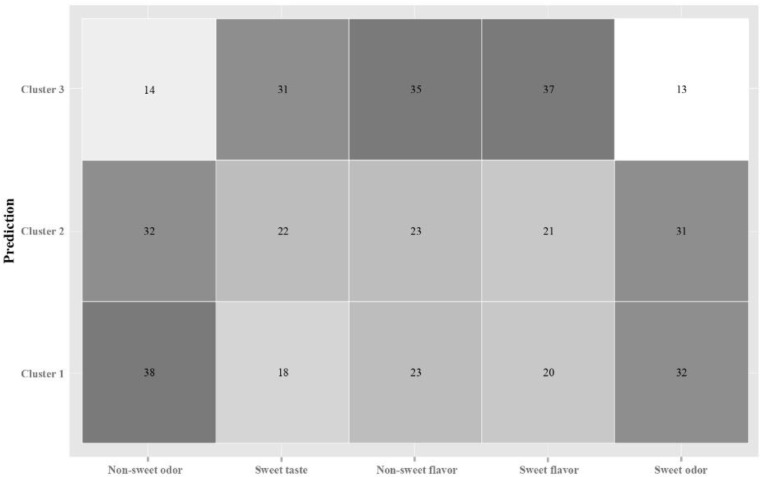
Confusion matrix of clusters and epochs.

To explore potential groups of individuals with common/dissimilar brain patterns for odor and taste/flavor classification, clustering techniques were first used to group by participants. Hierarchical clustering analysis showed three clusters with different brain responses to the samples. These clusters grouped the events of certain individuals that showed similar signals patterns for the samples. Cluster 2 (n = 4 individuals) was characterized by low standard deviation values for CP2, CP6, FP2, CP1, and PO7 and low maximum values in electrodes FC4, FPZ, and CP1. Cluster 1 (n = 6) had middle values, and cluster 3 (n = 8) higher values in these positions. Studying the relationship among clusters and epochs, the confusion matrix indicated that clusters 1 and 2 were closely related to odor stimuli discrimination, while cluster 3 was more related to taste and flavor discrimination (Figure 3). No further analyses were conducted within each of the clusters because of the limited data points and because studying individuals’ variability was not one of the aims of the research.

To assess the discrimination capacity of the group ‘odor’ from ‘taste/flavor’, a decision tree was calculated indicating both categories. The resulting decision tree, generated by the ‘rprt’ algorithm (recursive partitioning and regression trees; [29]), showed that variables CP6, CPZ, and CP4 were useful to differentiate taste/flavor from odor stimuli (in the mouth from orthonasal stimulation). The confusion matrix showed a great performance, with 88% balanced accuracy. Once odors had been discriminated from taste/flavors, a second decision tree was calculated to assess odor stimuli classification (orthonasal stimulation). PO6, POZ, and PO7 were found to be significant for ‘sweet-odor’ and ‘non-sweet odor’ discrimination (80% balanced accuracy). Finally, for taste/flavor discrimination (removing odor stimuli signals from the model), a higher number of electrodes was significant: C2, CP4, PO6, PZ, C6, CP5, PO7, and PO5, showing a higher complexity on the signal processing task (79% balanced accuracy). Both ‘non-sweet flavor’ (cayenne msm) and ‘sweet-flavor’ (vanilla msm) were accurately classified, while ‘sweet-taste’ discrimination (basic msm) was less precise. This result suggested that the presence of aromatic compounds together with the gustatory (flavor) stimuli eased the classification task.

**Figure 4:**
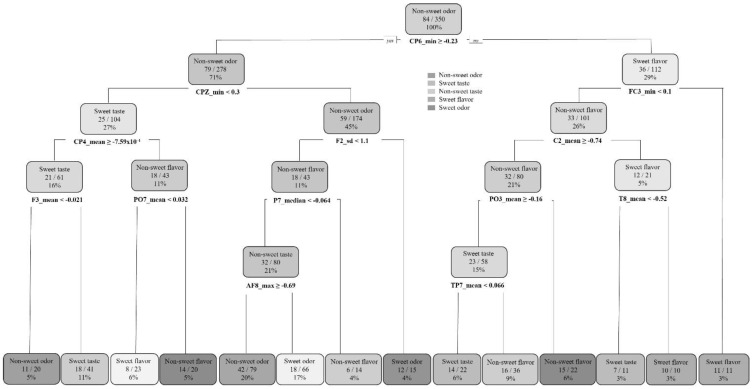
Decision tree of odor, taste, and flavor samples’ discrimination task.

The decision trees used to study the stimuli classification showed that CP6 was important to discriminate odor from taste/flavor; then, in the second level of the decision tree, CPZ seemed to be also relevant, suggesting that the parietal region of the brain could be associated with the odor and taste/flavor discrimination task (Figure 4).

**Figure 5:**
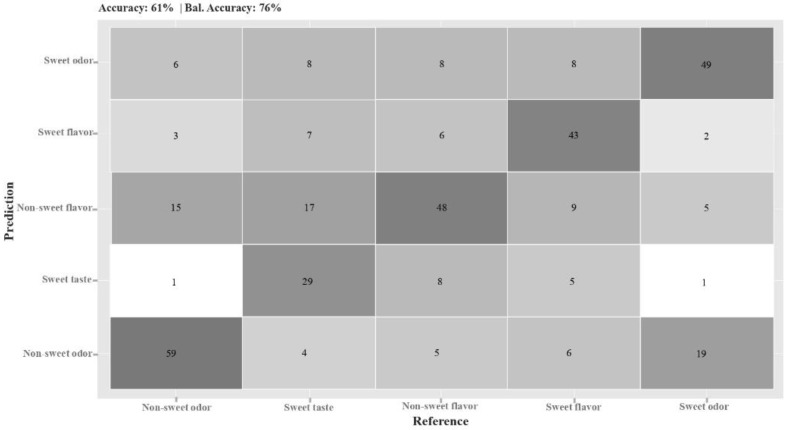
Confusion matrix of the descriptive model shown in Figure 4.

The confusion matrix associated with this whole descriptive model presented a 76% balanced accuracy (Figure 5). ‘Non-sweet odor’, ‘sweet-flavor’ (vanilla msm), and ‘non-sweet flavor’ (cayenne msm) were accurately classified by the model, although it was not as effective in distinguishing ‘sweet’ from ‘non-sweet flavor’.

When stimuli were grouped into ‘sweet’ and ‘non-sweet’, independently of their sensory modality (odor, taste, flavor), O1, FC6, FC1, and P7 were the locations significant for the discrimination of the different events, showing that the ‘sweet’ versus ‘non-sweet’ sensations were classified by different brain areas located at the temporal and parietal lobes. The performance of this last descriptive model was also useful, showing a 71% of balanced accuracy in the confusion matrix; due to being more general and including all stimuli, this model presented slightly lower accuracy than the more specific ones (e.g., the ‘sweet’ vs. ‘non-sweet’ odors one).

### 3.2. Explicit Response and Its Relationship with the Implicit Response

Results of the ANOVA test on the explicit response data showed that liking was significantly different for the samples (*p* < 0.05), being higher for the ‘sweet-odor’ sample (vanillin) than the ‘non-sweet odor’ and ‘non-sweet flavor’ samples (DMS and cayenne marshmallow). Samples with sweet taste and sweet flavors (marshmallows made with sucrose and vanillin) were not different from the rest of the samples (Table 1), suggesting that odor stimuli (orthonasal stimulation) could elicit stronger reactions. A similar pattern was observed for the emotional categories, with the ‘sweet-odor’ sample being significantly different than the ‘non-sweet odor’ and ‘non-sweet flavor’ samples for the ‘joyful’, ‘passionate’, ‘disgusted’, and ‘melancholic’ categories. The non-sweet samples, for both odor and flavor stimuli, only elicited higher responses on the ‘disgusted’ emotional category. The ‘sweet-flavor’ sample, characterized by a significant reduction of sucrose content (if compared with the sweet taste sample) and an addition of vanillin extract, was perceived as similar as the ‘sweet-taste’ sample for liking and all the emotional categories. The addition of the aroma did not elicit a different reaction, although the ‘sweet-odor’ sample with vanillin received higher scores for liking and the positive emotional categories.

**Figure 6:**
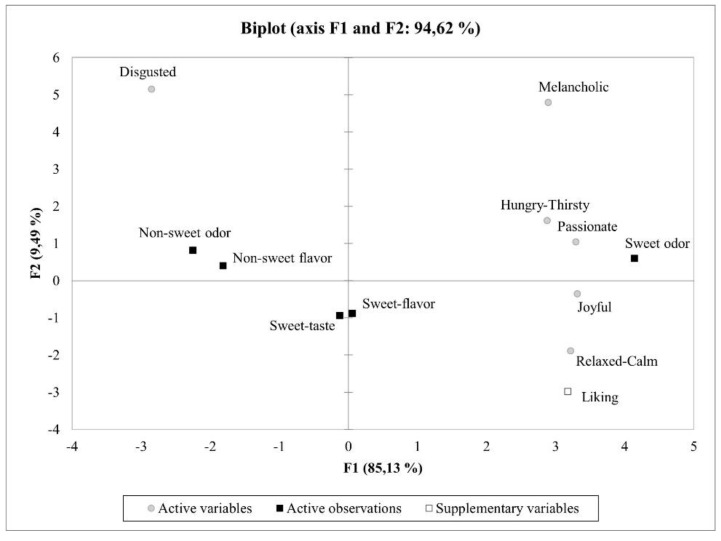
PC1 and PC2 biplot of the PCA (principal component analysis) showing the relationship among emotions elicited by the different samples (liking as supplementary variable).

Figure 6 shows a PCA biplot of the results from the explicit response. The first two principal components (PCs) explained 94.6% of the variance of the data. The ‘sweet-odor’ sample was positively correlated with liking and the positive emotions categories (e.g., joyful, passionate), whereas ‘non-sweet odor’ and ‘non-sweet flavor’ samples were positively correlated with the negative emotions of the ‘disgusted’ group.

To study the implicit–explicit responses relationship, an ANOVA test was conducted using ‘implicit cluster’ as factor, and the explicit responses as dependent variables. Only the ‘hungry’ category was significantly different for the three studied clusters, being significantly higher in cluster 1 than in cluster 3. These results should be considered tentative because of the different ‘n’ in each cluster (four, six, and eight subjects in clusters 1, 2, and 3, respectively).

## 4. Discussion

After the EEG analysis using artificial intelligence, it can be assumed that the EEG data patterns observed during the training phase followed a consistent pattern for all the participants. Therefore, the predictive model maintained its balanced accuracy when adding data from new subjects for its evaluation. In general, predictive models resulted as accurate to discriminate sensory modalities (odor–taste–flavor) and sweet from non-sweet stimuli. Andersen et al. [12] found that EEG responses allowed to discriminate perceptually similar sweet tastes and that the discrimination ability of the EEG data was related with the discrimination ability of the individual, but not related to calorie content. Although results of their research showed a similar grand-average evoked potential for the three tested sweet tastes (aspartame, sucrose, and a mixture of aspartame and acesulfame K), and therefore a similar brain response pattern for these substances, a within-participant analysis on a single-trial level allowed to discriminate among responses. Mouillot et al. [13] found different results, showing that sucrose, aspartame, and stevia generated differential brain responses (gustatory evoked potentials, GEP) mainly detectable in the P1 latencies (P1 being the first peak of the GEP) at PZ, CZ, FZ, FP1, and FP2 electrodes. Results of the present study showed some positions related to sweet odor and flavors classifications, some of them common with the ones reported by Mouillot et al. [13] and mainly located at the central and parietal areas of the brain (CP6, CPZ).

Results of the present research also showed a potential flow of information of the electrical signals through the brain when processing flavor and odor stimuli. A preliminary idea of the topological route of brain activity could be pictured following the different branches of the different decision trees, which showed the way that brain signals traveled (see Supplementary Material for reviewing all decision trees and confusion matrices). The inferred process of the analytic models showed that the brain channels CPZ, T7, and FT8 were the ones that provided a higher explanation of the variability of the data regarding the differentiation between odor and taste. Then, signals were discriminated in two different locations: the odor signals and its classification were at the rear areas of the brain (O1, O2, PO6), while taste and flavor were associated with the central areas (PO3, C3, C2, C4). Therefore, the process of events differentiation seemed to involve a sub-processing of information in different regions of the brain. Based on a topological analysis of the direction of the signals, it was possible to see how different individuals’ brains were working to classify the stimuli.

Crouzet et al. [14] showed that different taste neuronal response patterns, recorded using a 64-channel EEG, were related to taste recognition and discrimination (salty, sweet, sour, and bitter), and that this could, in turn, be related to perceptual decision-making. Small, Gerber, Mak, and Hummel [30] showed than the same odor may produce differential brain responses depending on whether it was experienced orthonasally or retronasally (in the mouth). In addition, Kroupi et al. [17] demonstrated that odor pleasantness could be predicted with EEG data, identifying mainly a subject-specific response and some generic patterns. Results from the present study suggested that some response patterns were related to specific sensory systems (odor, taste), similar to the taste recognition results showed by these authors when studying different tastes and odors. In addition, the present study showed other neuronal response patterns useful to predict sweet/non-sweet stimuli classification, independently of the stimulated sensory system. Small et al. [31] showed, using fMRI, that flavor perception and taste–smell integration was dependent on the olfactory delivery mode (orthonasal vs. in the mouth), and on the previous experience of the taste/smell combinations. Therefore, it is possible that similar recognition patterns are shown when an individual is exposed to different sensory stimuli that he/she has previously tasted together, and therefore grouped as a ‘sensory set’ (e.g., sweet taste with vanilla odor). Although different individuals’ clusters were identified in the present study, no further analyses were conducted in each of the clusters, because of the limited data points, and because it was not the specific aim of the research. Future research could explore the specific differences among clusters.

Different studies have reported EEG results to odor stimuli and its relationship with elicited emotions. Davidson [9] suggested that the frontal lobe activation could be associated with emotions and feelings, using mainly the alpha band (8–13 Hz). On the contrary, Martin [32] found theta activity to be related to attention/cognitive load when individuals were exposed to olfactory stimuli. The present research looked for similarities between liking and the emotional self-reported response to the implicit response recordings, but no relationship was identified between explicit and implicit responses. EEG results seemed to be related to the odor and flavor discrimination/recognition task and not to the self-reported emotional response, and clusters of individuals were not associated with the emotional response to the samples (ANOVA results using cluster as factor). A similar pattern on the liking response and the ‘sweet’ vs. ‘non-sweet’ implicit response could be intuited, but a higher number of responses would be needed to verify this trend.

The present study contributes to consumer neuroscience research in providing new insights about odor and flavor stimuli brain processing, although extensive research is needed to clarify if the implicit data was strictly related to the different sensory systems activation or to the quality of the stimuli. Taste quality is among the first attributes represented in the central gustatory system and detectable by EEG recordings [14], and olfactory stimuli have been associated by different authors with activation/reduction of brain activity, e.g., [32,33], but food stimuli are complex and difficult to study for activating independent sensory systems. One of the limitations of the present study lies on this complexity, considering the difficulty of designing a food matrix in which texture would not significantly interfere with the aroma and taste release, or choosing the most appropriate non-sweet stimuli (e.g., cayenne pepper causes irritation and can activate more than gustatory and olfactory receptors). Niedziela and Ambroze [34] recently reported the complexity of food neuroscience research, pointing out the need for a multidisciplinary team to collect and analyze data. Further research is needed to propose new methodologies and data analysis strategies to easy quality research development in this young field. De Wijk and Noldus [35] suggested using implicit measurements in real context to capture the total food experience from pre- to post-consumption, including, therefore, the physical and social context impact on food perception. With that aim in mind, further studies should be conducted to better understand the real nature of the collected data and their relationship with food perception and food-related decisions. Including placebos or blank samples to emulate the different sensory modalities when studying a particular one (e.g., using a non-tasty marshmallow when assessing an odorant in the present research) could improve the experimental design by better mimicking the experience of eating. In addition, segmenting the signal by frequency band to build the model, instead of using the whole amplitude, could provide additional data on the importance of each of the frequencies to discriminate events.

## 5. Conclusions

EEG responses were recorded for different sweet and non-sweet stimuli belonging to odor, taste, and flavor sensory modalities. Significant differences were found among samples’ liking and the emotions elicited by the samples, and artificial intelligence tools (‘rprt’ algorithm) allowed to classify stimuli by activated sensory system (odor/taste/flavor) and nature of the stimuli (‘sweet’ vs. ‘non-sweet’ odors; ‘sweet-taste’, ‘sweet-flavor’ and ‘non-sweet flavor’; ‘sweet stimuli’ vs. ‘non-sweet stimuli’), but no clear relationship was detected between explicit and implicit data. Further research is needed to clarify if the data provided by the EEG recordings were somehow related to the specific quality of the stimuli, to the task of recognizing and categorizing some stimuli to which respondents were familiar, or to the way than the stimuli are detected, because ‘in-the-mouth’ activation will elicit the triggering of tactile receptors, salivary glands, etc. Despite the limitations of the present study, the presented results provide a first step in studying the relationship between odor, taste, and flavor stimuli with a common descriptor⁠—sweetness—and could serve as a starting point in cross-modal implicit sensory research.

## Figures and Tables

**Table 1:** ANOVA results for the liking and the 6 emotion categories in the 5 samples. Legend: different letters within the same row indicate significant differences by Tukey’s HSD (*p* < 0.05).

Acceptance/Emotion Category	Sample					*p*-Value
	Sweet-Odor (Vanillin)	Sweet-Flavor (Vanillin)	Sweet-Taste (Sucrose)	Non-Sweet Flavor (Cayenne)	Non-Sweet Odor (DMS)	
Liking	7.5 a	6.2 ab	6.1 ab	4.5 b	4.3 b	<0.05
Joyful	10.0 a	6.7 ab	6.1 ab	4.3 b	4.4 b	<0.05
Passionate	9.2 a	4.6 b	5.0 ab	3.1 b	3.4 b	<0.05
Disgusted	1.3 c	2.7 bc	3.1 bc	6.9 ab	8.8 a	<0.001
Hungry/thirsty	6.8	4.2	3.9	4.8	2.7	0.170
Melancholic	6.1	3.1	2.9	2.8	3.5	0.145
Relaxed/calm	8.8 a	5.4 ab	5.8 ab	2.3 b	2.9 b	<0.001

## Data Availability

The data that support the findings of this study are available from the corresponding author upon reasonable request.

## Supplementary Materials

The following supporting information can be downloaded at: https://www.mdpi.com/article/10.3390/s22186787/s1. Figure S1a. Decision tree of odor vs. taste/flavor discrimination task.; Figure S1b. Confusion matrix of the descriptive model shown in Figure S1a; Figure S2a. Decision tree of sweet odor vs. non-sweet odor discrimination task; Figure S2b. Confusion matrix of the descriptive model shown in Figure S2a.; Figure S3a. Decision tree of taste/flavor discrimination task (sweet taste, sweet flavor, and non-sweet flavor); Figure S3b. Confusion matrix of the descriptive model shown in Figure S3a; Figure S4a. Decision tree of sweet (sweet odor, sweet taste & sweet flavor) vs. non-sweet (non-sweet odor and non-sweet flavor) discrimination task; Figure S4b. Confusion matrix of the descriptive model shown in Figure S4.
