# Supplementary material for: Exploratory Research on Sweetness Perception: Decision Trees to Study Electroencephalographic Data and Its Relationship with the Explicit Response to Sweet Odor, Taste, and Flavor
Source: Sensors (Basel). 2022 Sep 8;22(18):6787. doi: 10.3390/s22186787 (PMC9504051; doi:10.3390/s22186787)
Supplement: Supplementary file 1 [file sensors-22-06787-s001.zip › sensors-1821617-supplementary.pdf]

SUPPLEMENTARY MATERIAL

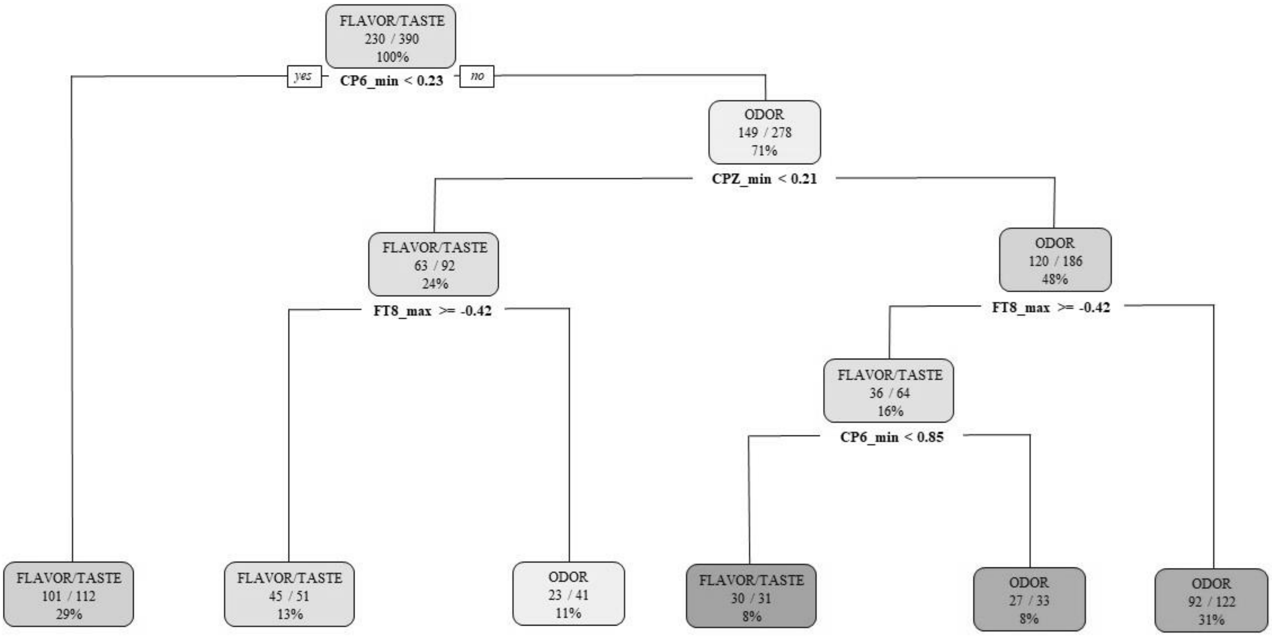

Figure S1a. Decision tree of odor *vs*. taste/flavor discrimination task.

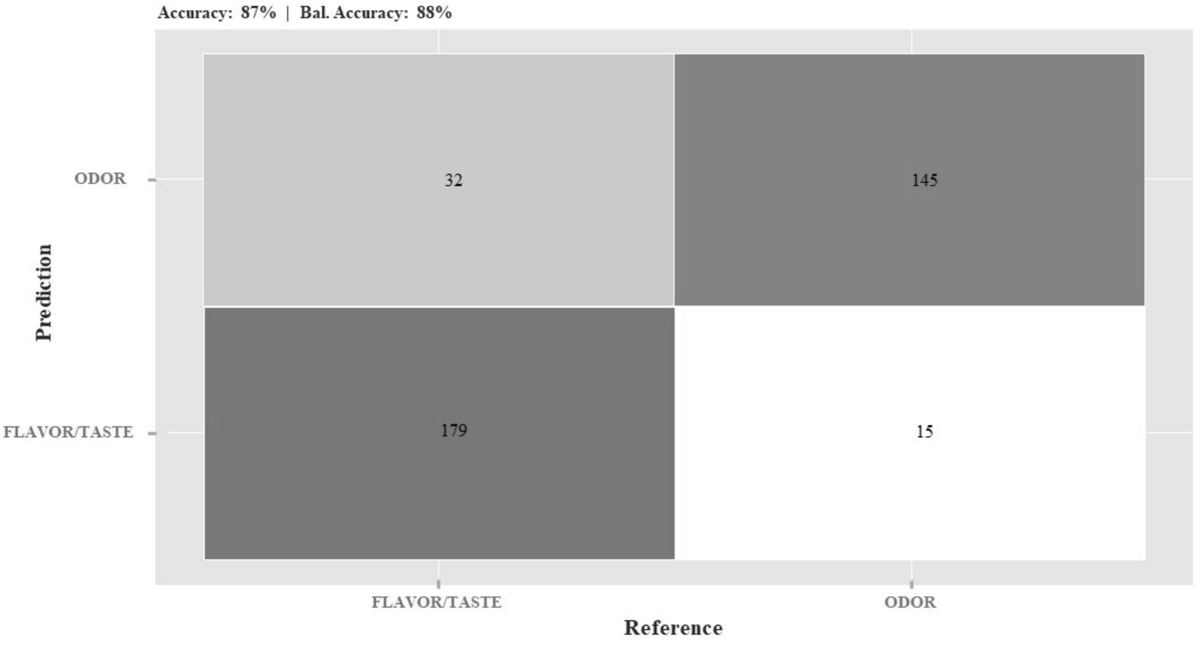

Figure S1b. Confusion matrix of the descriptive model shown in Figure S1a.

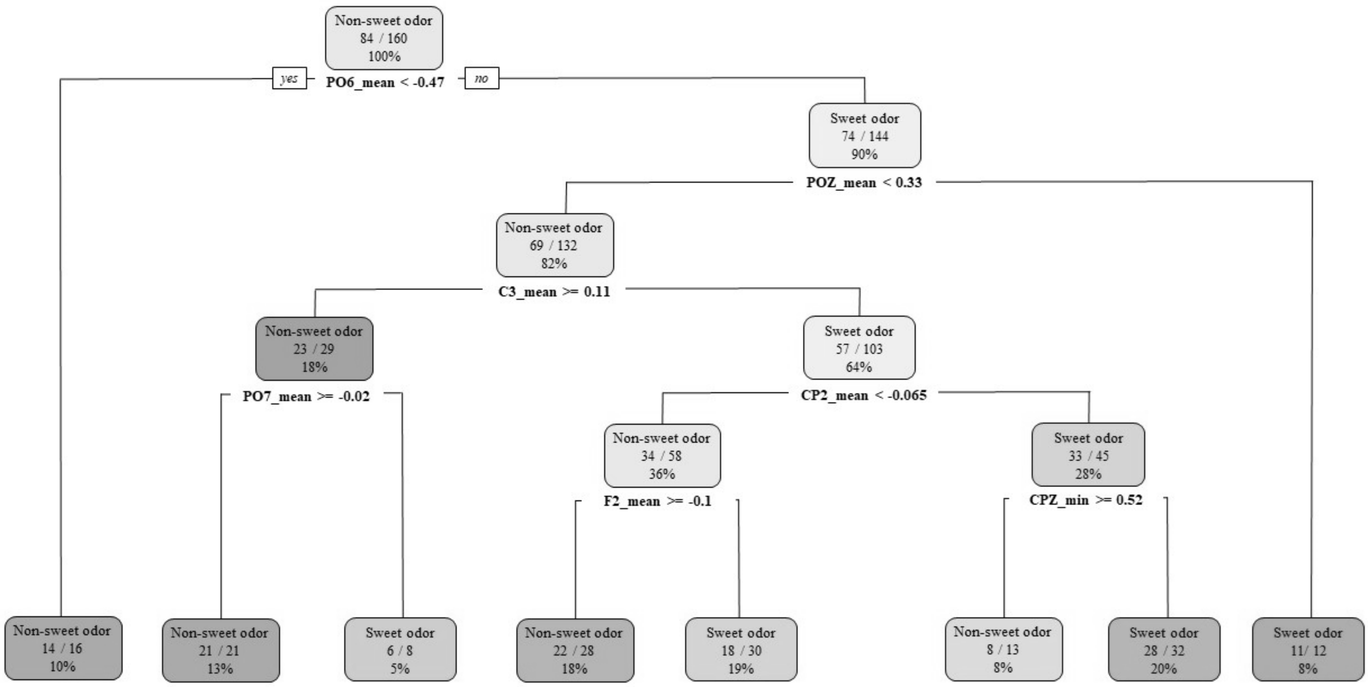

Figure S2a. Decision tree of sweet odor *vs.* non-sweet odor discrimination task.

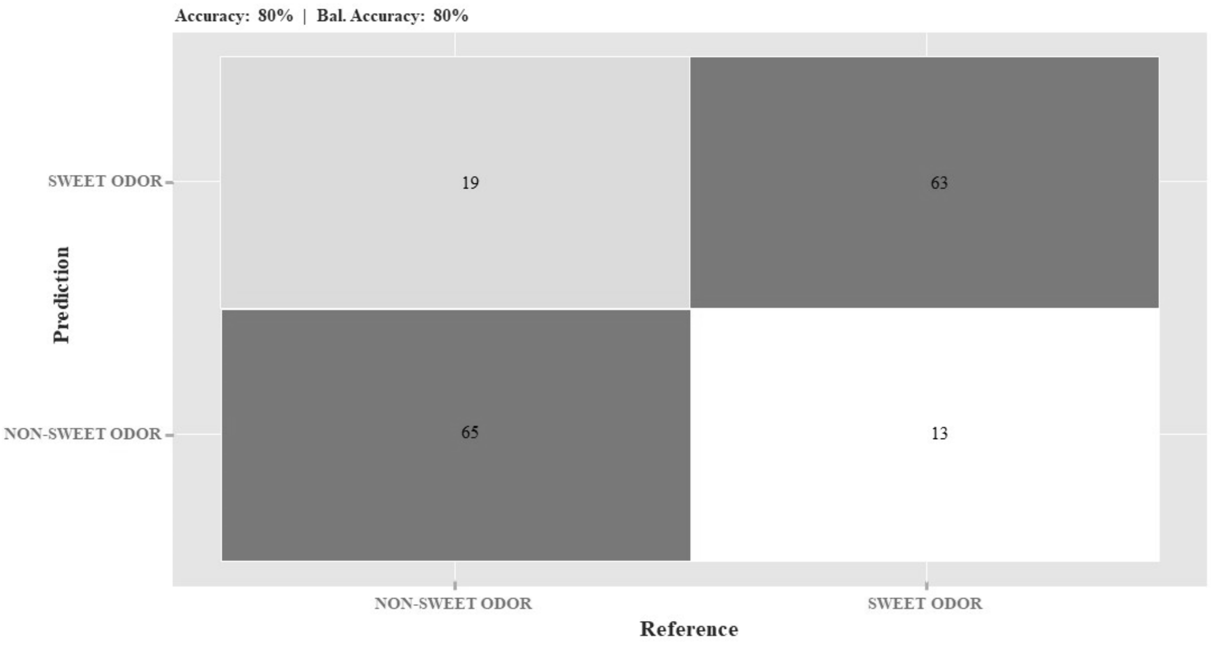

Figure S2b. Confusion matrix of the descriptive model shown in Figure S2a.

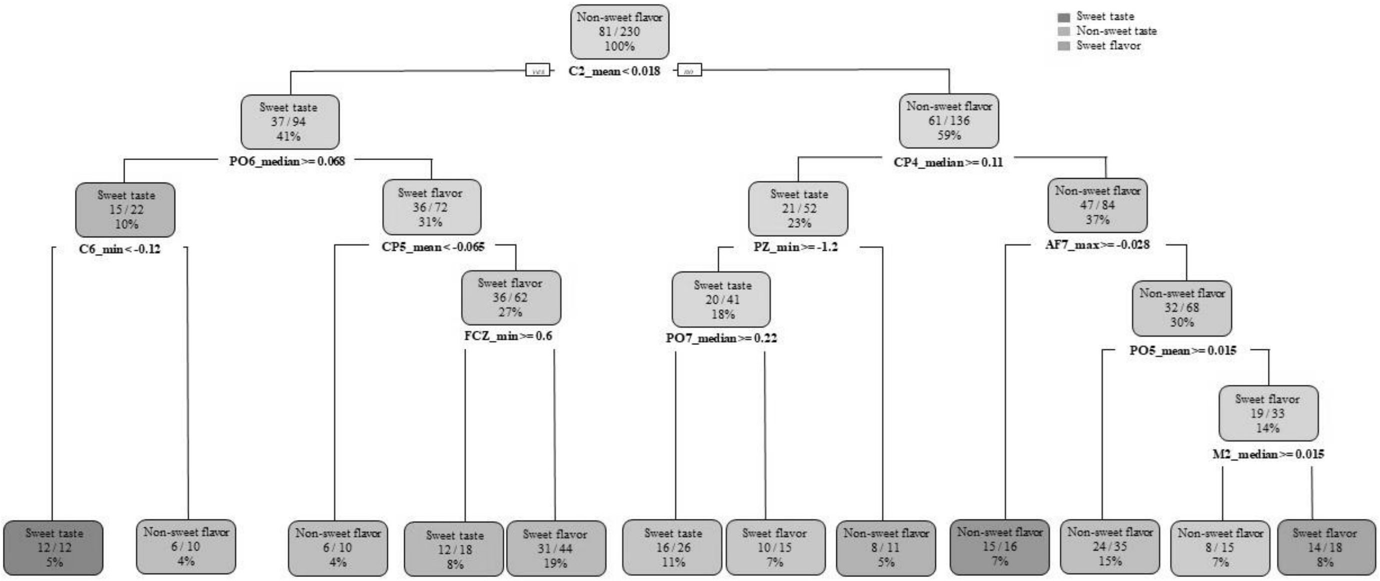

Figure S3a. Decision tree of taste/flavor discrimination task (sweet taste, sweet flavor & non-sweet flavor).

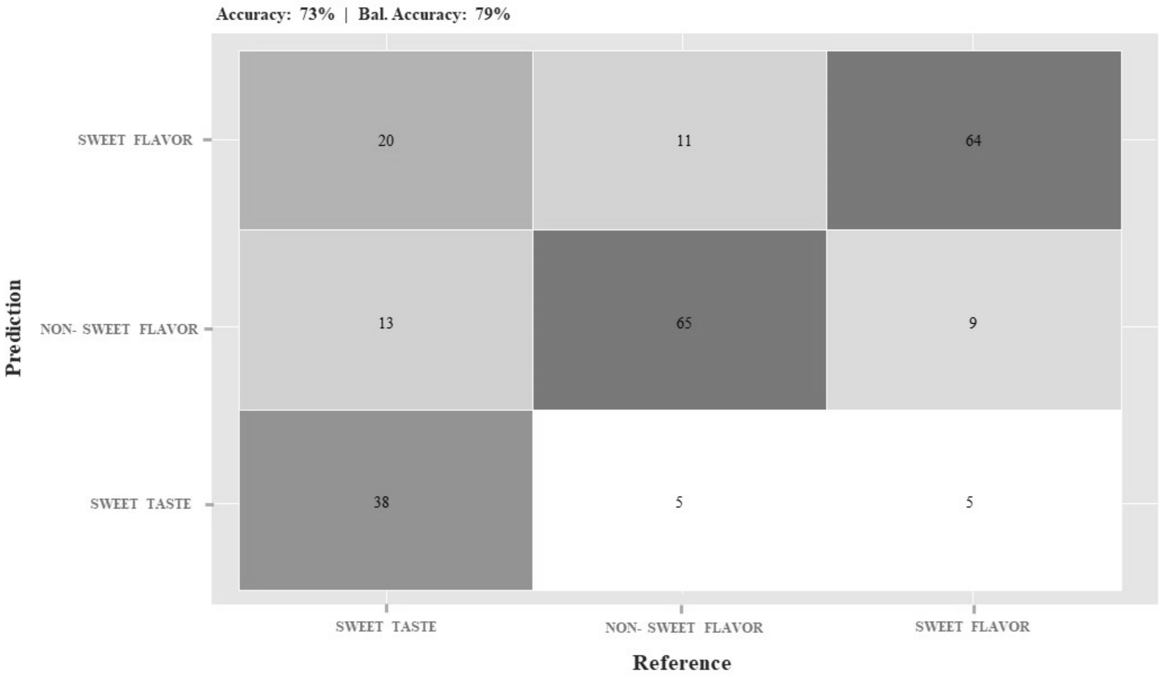

Figure S3b. Confusion matrix of the descriptive model shown in Figure S3a.

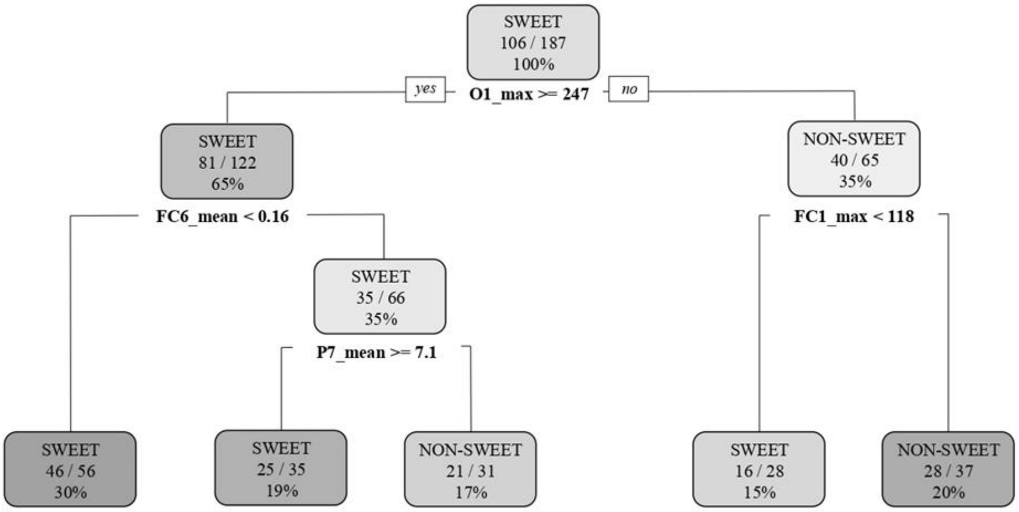

Figure S4a. Decision tree of sweet (sweet odor, sweet taste & sweet flavor) *vs.* non-sweet (non-sweet odor & non-sweet flavor) discrimination task.

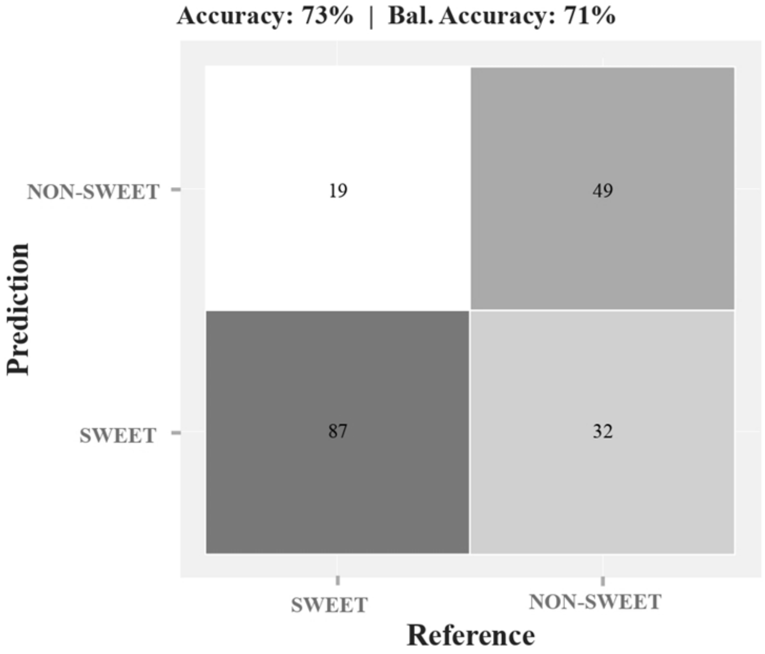

Figure S4b. Confusion matrix of the descriptive model shown in Figure S4a.
